# Update on the Angiotensin Converting Enzyme 2-Angiotensin (1–7)-Mas Receptor Axis: Fetal Programing, Sex Differences, and Intracellular Pathways

**DOI:** 10.3389/fendo.2013.00201

**Published:** 2014-01-09

**Authors:** Mark C. Chappell, Allyson C. Marshall, Ebaa M. Alzayadneh, Hossam A. Shaltout, Debra I. Diz

**Affiliations:** ^1^The Hypertension and Vascular Research Center, Wake Forest University School of Medicine, Winston-Salem, NC, USA; ^2^Department of Obstetrics and Gynecology, Wake Forest University School of Medicine, Winston-Salem, NC, USA; ^3^Department of Pharmacology and Toxicology, School of Pharmacy, Alexandria University, Alexandria, Egypt

**Keywords:** Ang-(1–7), Ala^1^-Ang-(1–7), ACE2, ACE, Mas receptor, Mas-related receptor D, fetal programing

## Abstract

The renin-angiotensin-system (RAS) constitutes an important hormonal system in the physiological regulation of blood pressure. Indeed, dysregulation of the RAS may lead to the development of cardiovascular pathologies including kidney injury. Moreover, the blockade of this system by the inhibition of angiotensin converting enzyme (ACE) or antagonism of the angiotensin type 1 receptor (AT_1_R) constitutes an effective therapeutic regimen. It is now apparent with the identification of multiple components of the RAS that the system is comprised of different angiotensin peptides with diverse biological actions mediated by distinct receptor subtypes. The classic RAS can be defined as the ACE-Ang II-AT_1_R axis that promotes vasoconstriction, sodium retention, and other mechanisms to maintain blood pressure, as well as increased oxidative stress, fibrosis, cellular growth, and inflammation in pathological conditions. In contrast, the non-classical RAS composed of the ACE2-Ang-(1–7)-Mas receptor axis generally opposes the actions of a stimulated Ang II-AT_1_R axis through an increase in nitric oxide and prostaglandins and mediates vasodilation, natriuresis, diuresis, and oxidative stress. Thus, a reduced tone of the Ang-(1–7) system may contribute to these pathologies as well. Moreover, the non-classical RAS components may contribute to the effects of therapeutic blockade of the classical system to reduce blood pressure and attenuate various indices of renal injury. The review considers recent studies on the ACE2-Ang-(1–7)-Mas receptor axis regarding the precursor for Ang-(1–7), the intracellular expression and sex differences of this system, as well as an emerging role of the Ang1-(1–7) pathway in fetal programing events and cardiovascular dysfunction.

## Introduction

Over the past 20 years the concept of the renin-angiotensin-system (RAS) as a monolithic endocrine system reflected primarily by the interaction of the peptide Angiotensin II (Ang II) with the AT_1_-receptor subtype has undergone extensive revision. The emerging view of alternative pathways within the RAS that may functionally antagonize the Ang II-AT_1_-receptor axis may be traced back to both the characterization of the AT_2_ receptor subtype and the identification of the heptapeptide des-[Phe^8^]-Angiotensin II or Angiotensin-(1–7) [Ang-(1–7)] in the circulation and various tissues (1–4). Since that time, the elaboration of distinct biochemical components that comprise the “Ang-(1–7) axis” is now firmly established with the identification of a unique receptor for Ang-(1–7) – the G-protein coupled Mas receptor, selective antagonists and agonists for the receptor, and an angiotensin II converting enzyme (ACE2) that catalyzes the processing of Ang II to Ang-(1–7) (5–9). In addition to the identification of the components of the Ang-(1–7) system, there is the recognition of various signaling pathways including nitric oxide (NO), prostaglandins, and cellular phosphatases that are stimulated by the peptide (10, 11). Although the early studies of Ang-(1–7) primarily sought to establish a role for Ang-(1–7) in the regulation of blood pressure, particularly as endogenous levels of the peptide increase markedly following angiotensin converting enzyme (ACE) or AT_1_-receptor blockade; the pressure-independent actions of the Ang-(1–7) axis should be considered with perhaps equal importance (6, 12, 13). Indeed, the beneficial actions of Ang-(1–7) system encompass various pathologies from cancer and the anti-proliferative actions of the peptide to diabetes and the cellular effects on stem cells (8, 9, 14). In turn, deficiencies in Ang-(1–7) that contribute to autonomic dysfunction were apparent in hypertension (15) and aging (16); Ang-(1–7) deficiency in hypertension was restored by ACE inhibitor treatment (17) or chronic Ang-(1–7) replacement (18). The breadth of these effects is not surprising as the RAS is a tissue system whose protein and peptide components are expressed in essentially every organ and whose actions are implicated in numerous physiological events that influence renal, neuronal, cardiac, pancreatic, vascular, adrenal, pituitary, cognitive, aging, inflammatory, and reproductive functions (19). As the Ang II-AT_1_-receptor axis of the RAS is increasingly recognized as a key regulatory pathway in various tissues and cells, the counter-balancing Ang-(1–7) axis should be evident as well. In this review, we consider the current literature on the ACE2-Ang-(1–7)-Mas receptor axis regarding the sources for Ang-(1–7), the intracellular expression of this system, the emerging role of Ang1-(1–7) pathway in fetal programing events and cardiovascular dysfunction, and finally, the evidence for sex-dependent regulation and function of the Ang-(1–7) axis.

## Sources of Angiotensin-(1–7)

### Endopeptidases

**Figure 1:**
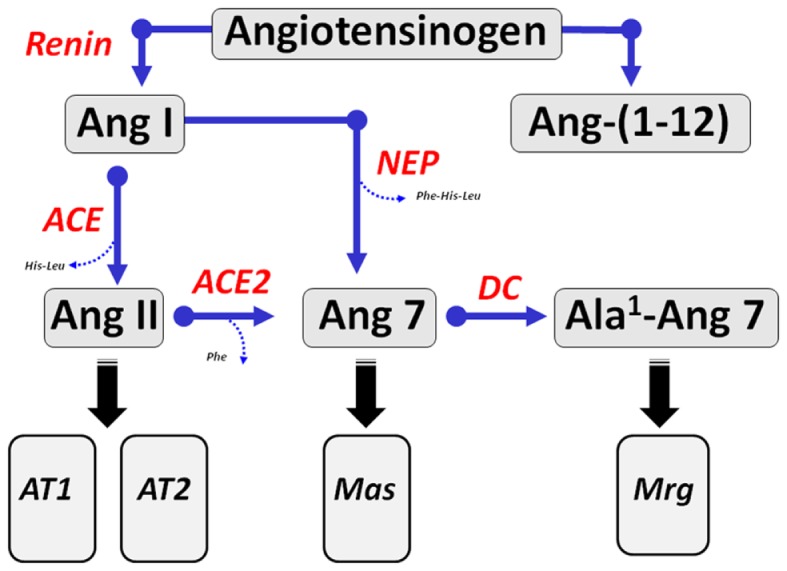
**Enzymatic cascade of angiotensin peptide formation and metabolism**. Renin cleaves the precursor protein angiotensinogen to angiotensin-(1–10) (Ang I) which is further processed to the biologically active peptides Ang-(1–8) (Ang II) by angiotensin converting enzyme (ACE) and Ang-(1–7) by endopeptidases such as neprilysin (NEP). Ang II undergoes further processing at the carboxyl terminus by the carboxypeptidase ACE2 to yield Ang-(1–7) (Ang 7). Ang-(1–7) undergoes decarboxylation (DC) of the aspartic acid residue to form Ala^1^-Ang-(1–7) (Ala^1^-Ang 7). The dodecapeptide Ang-(1–12) is derived from the hydrolysis of the Tyr^12^-Tyr^13^ bond of rat angiotensinogen by an unknown enzymatic pathway. Ang II recognizes both AT_1_ and AT_2_ receptors. Ang-(1–7) activates the Mas receptor and Ala^1^-Ang-(1–7) recognizes the Mas-D related receptor (Mrg).

Angiotensinogen, a glycosylated protein that is primarily synthesized and secreted by the liver as well as other tissues is the sole precursor for angiotensin peptides (20). The only known substrate for the aspartyl protease renin is angiotensinogen which releases the decapeptide Ang I from the amino-terminal portion of the protein (Figure 1). Ang I is then cleaved by ACE to form the bioactive peptide Ang II. Early studies revealed that endogenous levels of both Ang I and Ang-(1–7) were markedly increased following the administration of ACE inhibitors (21, 22). The augmented response in Ang-(1–7) suggested that the circulating peptide may contribute to the beneficial actions of the inhibition of ACE pathway in addition to that of reducing endogenous levels of Ang II. The increase in Ang-(1–7) in the presence of ACE blockade necessitates a processing pathway independent of the formation of Ang II. Several studies subsequently showed that the endopeptidase 3.4.24.11 (neprilysin) contributed to the circulating levels of Ang-(1–7) in animals chronically treated with various ACE inhibitors (23–26). Ang I infusion in normotensive WKY and hypertensive spontaneously hypertensive rat (SHR) treated with the ACE inhibitor lisinopril resulted in higher plasma levels of Ang-(1–7) and co-administration of the neprilysin inhibitor SCH39370 but not the prolyl oligopeptidase (POP) inhibitor *z*-prolyl prolinal abolished the circulating levels of the peptide (27). Moreover, acute infusion of a similar dose of Ang II did not increase circulating Ang-(1–7) in either control or lisinopril-treated WKY and SHR. The increase in Ang-(1–7) following ACE blockade reflects both a reduction in Ang-(1–7) metabolism and alternative processing of Ang I through tissue-specific endopeptidases (23). In this regard, Pereira et al. recently demonstrated that the endopeptidase EC3.4.24.15 (thimet oligopeptidase) may contribute to formation of Ang-(1–7) in the rat hippocampus (28). Interestingly, these investigators reported higher expression of this peptidase and the Mas receptor in a rat model of temporal lobe epilepsy suggesting a possible role of the Ang-(1–7)-Mas axis in this central pathology (28). Indeed, the study supports earlier reports of the direct processing of Ang I to Ang-(1–7) by thimet oligopeptidase in vascular smooth muscle cells and a rat hindlimb perfusion system (29, 30).

### ACE2

Apart from endopeptidases that process Ang I or Ang-(1–12) to Ang-(1–7), various mono-carboxypeptidases including prolyl carboxypeptidase (PCP), POP, and the ACE homolog ACE2 generate Ang-(1–7) directly from Ang II. It should be emphasized that PCP requires an acidic pH optimum for activity, but may contribute to lysosomal pathways for metabolism of internalized Ang II or to the processing of Ang II to Ang-(1–7) in urine (31). ACE2 continues to be of primary focus given its ability to effectively metabolize Ang II and generate Ang-(1–7) (32, 33). ACE2 exhibits an efficiency constant (*V*_max_/*k*_M_ or *k*_cat_) for Ang II that is 10- to 100-fold higher than that of PC or POP. In this regard, the soluble form of ACE2 has been utilized as a therapeutic agent to reduce blood pressure and attenuate target organ damage in hypertensive and diabetic animal models (34–37). ACE2 mRNA expression was increased in the brain medulla following long-term AT1-receptor blockade (38). It is unclear whether the beneficial effects of ACE2 administration reflect the reduction in Ang II, the enhanced formation of Ang-(1–7) or the increased ratio of Ang-(1–7) to Ang II. Moreover, Turner and colleagues report that soluble ACE2 attenuated the integrin-dependent stimulation of focal adhesion kinase (FAK) and increased the expression of the Akt kinase suggesting the peptidase may have direct cellular effects apart from its peptidase activity (39).

In addition to the functional role of ACE2 that catalyzes the conversion of Ang II to Ang-(1–7), the peptidase may serve as a biomarker of renal and cardiac pathologies. Two studies in type I (streptozotocin-induced) and type II (db/db mice) diabetic models reported an early increase in the urinary excretion of ACE2 (40, 41). The enhanced excretion of ACE2 in db/db mice closely correlated to the increase in albuminuria or proteinuria. Moreover, chronic treatment with insulin-sensitizing agent rosiglitazone improved the metabolic balance in the db/db mice and reduced the excretion of both ACE2 and albumin (40). In contrast to the reduction in urinary levels of ACE2, the increased renal expression of ACE2 in the db/db mouse was not altered by rosiglitazone which may reflect an added therapeutic benefit to maintain the peptidase in the diabetic kidney (40). An important aspect of the two latter studies suggests that in the diabetic kidney, the development of tissue injury should not necessarily be interpreted as arising from a deficit in ACE2 expression. Indeed, the increase in tissue and urinary levels of ACE2 in pathological conditions may reflect a compensatory response to alter the balance of Ang II and Ang-(1–7) pathways within a particular tissue or cell type (7). In this regard, the deleterious effects of an ACE2 inhibitor or knockdown of the enzyme may be particularly evident under conditions of enhanced ACE2 expression. The circulating levels of ACE2, which are typically low to not detectable, are also increased in experimental conditions of diabetes. We show in a model of diabetic hypertension that circulating ACE2 activity increased over fivefold in female mRen2.Lewis rats (42). However, serum ACE activity also increased suggesting that the potential beneficial effects of higher ACE2 may be offset by ACE acting to increase Ang II and metabolize Ang-(1–7). Indeed, plasma levels of Ang-(1–7) were not changed in the diabetic mRen2.Lewis despite the marked increase in ACE2 activity. Moreover, circulating ACE activity was substantially higher than that of ACE2 when assessed under similar incubation and substrate conditions for each enzyme (42).

**Figure 2:**
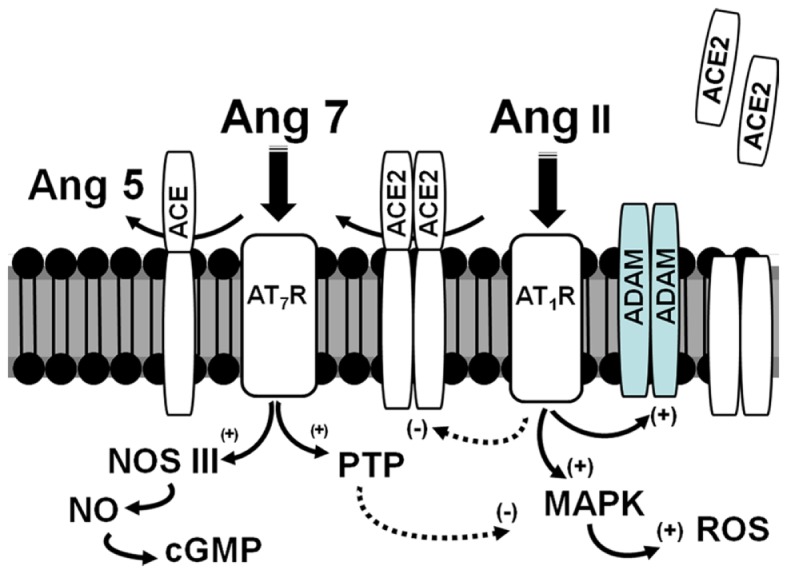
**Scheme for the interaction of ACE2 and ADAMs on the apical surface of the proximal tubules in the diabetic kidney**. Ang II binds to the AT_1_ receptor (AT_1_R) and stimulates MAP kinase (MAPK) pathways and production of reactive oxygen species (ROS). The Ang II-AT_1_-receptor axis may attenuate ACE2 expression but increase ADAM levels. ACE2 is anchored to the apical membrane and directly converts Ang II to Ang-(1–7) (Ang 7); ACE also anchored to the membrane metabolizes Ang-(1–7) to Ang-(1–5) (Ang 5). Ang-(1–7) recognizes the AT_7_/Mas receptor (AT_7_R) to antagonize the actions of the Ang II-AT_1_R by stimulation of protein phosphatases (PTP) and nitric oxide synthase (NOS) to form nitric oxide (NO) and cGMP. In pathological conditions, increased expression of ADAMs may hydrolyze ACE2 away from the apical surface to increase local concentrations of Ang II and reduce the levels of Ang-(1–7).

In the db/db mice, infusion of exogenous ACE2 that markedly increased serum levels of the enzyme did not alter urinary ACE2 suggesting that the enzyme is not readily filtered by the glomerulus (41). One mechanism for the increase in urinary excretion of ACE2 is the regulated shedding of the enzyme from the apical face of the proximal tubules (Figure 2). Studies by Lambert and colleagues originally reported that the disintegrin and metalloproteinase (ADAM17) secretase was responsible for the release of ACE2 (43). A subsequent report identified a specific sequence of the juxtamembrane stalk of ACE2 hydrolyzed that was by ADAM17 to release the peptidase from human pulmonary epithelial cells (44). In proximal epithelial cells of the db/db mouse kidney, there was extensive overlap of ACE2 and ADAM17 immunostaining (40). Moreover, rosiglitazone treatment attenuated ADAM17 expression which may contribute to the reduced shedding of ACE2 into the tubular fluid and subsequent excretion in the urine. In addition to the shedding of ACE2, ADAM17 may influence tissue damage by the release of the tethered inflammatory factors TNFα, EGF, and TGFα that subsequently activate their respective receptors in an autocrine or paracrine manner (45). If expression of ACE2 on the apical membrane of the tubules contributes to the regulation of the local concentrations of Ang II, an increase in ADAM17 may lead to inflammatory and fibrotic events through enhanced Ang II-AT_1_-receptor signaling, as well as increased cytokine and growth factor activation (Figure 2). Lazartigues and colleagues report that knockdown of ADAM17 in the brain of DOCA-salt mice reduced blood pressure, and increased the tissue expression of ACE2 (46). In this model of neurogenic hypertension, the benefit of ADAM17 knockdown may reflect a reduction of Ang II in brain; however, the direct effects on the release of EGF and other cytokines cannot be discounted. Indeed, the transactivation of the EGF receptor (EGFR) and signaling pathways is a key signaling event of the Ang II-AT_1_-receptor pathway (47). The increased shedding of ACE2 may also reduce levels of Ang-(1–7) and attenuate the inhibitory actions on the Ang II-AT_1_-receptor axis and other pro-inflammatory and pro-fibrotic pathways. Akhtar et al. recently reported that Ang-(1–7) attenuated EGFR activation in response to Ang II, as well as reduced the extent of renal injury in the diabetic SHR (48). Moreover, increasing evidence suggests that one of the primary pathways activated by Ang-(1–7) is the stimulation of various cellular phosphatases (PTP) including SHP-1 and DUSP-1 that may attenuate activated kinase-dependent pathways (49–53) (Figure 2).

### Angiotensin-(1–12)

Nagata and colleagues identified a novel endogenous angiotensin peptide termed Ang-(1–12) that contains the first 12 amino acids of the N-terminal sequence of rat angiotensinogen (Asp^1^-Arg^2^-Val^3^-Tyr^4^-Ile^5^-His^6^-Pro^7^-Phe^8^-His^9^-Leu^10^-Leu^11^-Tyr^12^) (54) (Figure 1). These investigators developed antibodies directed to the amino- and carboxyl-terminal sequences of Ang-(1–12) and demonstrated expression of Ang-(1–12) in essentially all tissues that contain Ang II with the highest levels in the intestine, brain, heart, plasma, and kidney of rat. Differential expression of Ang-(1–12) was evident in the heart and kidney of the SHR and the normotensive control Wistar Kyoto strain (WKY) (55). An antibody specific to the C-terminal sequence of rat Ang-(1–12) including Leu^11^-Tyr^12^ revealed selective staining in cardiac myocytes and proximal tubule cells of the kidney. The site of hydrolysis for formation of Ang-(1–12) from rat angiotensinogen occurs at residues Tyr^12^-Tyr^13^ which is distinct from the Leu^10^-Leu^11^ sequence cleaved by renin to form Ang I. Thus, the generation of Ang-(1–12) is likely through a non-renin dependent pathway and may be apparent in conditions of low or suppressed renin activity, particularly with the use of selective renin inhibitors. Similar to Ang I, Ang-(1–12) can be hydrolyzed at the Phe^8^-His^9^ bond by ACE or chymase to form Ang II (54, 56, 57). The conversion of Ang-(1–12) to Ang II by ACE in the circulation is consistent with the acute increase in blood pressure following an infusion of Ang-(1–12) in normotensive rats, as well as the blockade of the pressor response by either an ACE inhibitor or AT_1_-receptor antagonist. Arnold et al. also find that central Ang-(1–12) administration attenuated baroreflex sensitivity and the response was blocked by either an ACE inhibitor or AT_1_-receptor antagonist (57). Moreover, neutralization of Ang-(1–12) by intracerebroventricular (ICV) infusion of an affinity-purified antibody reduced blood pressure in the (mRen27)2 hypertensive rats consistent with the biochemical and immunocytochemical evidence for Ang-(1–12) in the rat brain (58). To our knowledge, the latter study by Isa and colleagues is the only report to date that demonstrates an endogenous role for Ang-(1–12).

As to the Ang-(1–7) axis, we recently demonstrated that Ang-(1–12) may be an alternative substrate for the generation Ang-(1–7) in the kidney (59). Isolated cortical membranes from the kidney of the hypertensive mRen2.Lewis rat processed Ang-(1–12) to Ang-(1–7) and Ang-(1–4). We observed a similar pattern of metabolism using the recombinant forms of mouse and human neprilysin. The selective neprilysin inhibitor SCH39370 abolished the formation of Ang-(1–7). We noted a peak corresponding to Ang I in the processing of Ang-(1–12) by the cortical membranes that was also abolished by the neprilysin inhibitor suggesting the peptide may be an intermediate in the processing of Ang-(1–12) to Ang-(1–7) (59). In these studies, we also show that circulating or renal renin did not metabolize Ang-(1–12) particularly in the presence of the ACE inhibitor lisinopril which implies that the peptide lacks the minimal sequence for recognition by renin (59). Bujak-Gizycka and colleagues demonstrated the generation of Ang-(1–12) in rat aorta homogenates by a serine peptidase using Ang-(1–14) as the substrate; however, the extent that this activity will process the angiotensinogen protein to Ang-(1–12) is not currently known (60). We did not detect the conversion of Ang-(1–12) to Ang-(1–7) in serum which would be consistent with the lack of soluble forms of neprilysin in the circulation, nor were there significant levels of Ang-(1–11) suggesting the absence of processing by ACE2 or other carboxypeptidases (59). It is feasible that Ang-(1–12) may be a potential substrate for Ang-(1–7) through the initial conversion to Ang-(1–11) by ACE2 and subsequent processing to Ang-(1–9) and Ang-(1–7) by ACE. However, ACE activity is far higher in the circulation than ACE2 and Ang-(1–7) formation from Ang-(1–12) or Ang I more likely reflects endopeptidase activity. Although further studies are required to discern the endogenous pathways for the formation and processing of Ang-(1–12), the peptide constitutes a potential substrate for the conversion to either the active products Ang II or Ang-(1–7).

### Ala^1^-Angiotensin-(1–7) and Pro^1^-Glu^2^-Ang II

In addition to the precursors to Ang-(1–7), the peptide itself may serve as a precursor to other active forms. Santos and colleagues recently identified an endogenous analog of Ang-(1–7) in which the aspartic acid residue was decarboxylated to alanine (Ala) forming Ala^1^-Ang-(1–7) (Figure 1) (61). The Ala^1^-Ang-(1–7) analog (also termed almandine) may also potentially arise from the proteolytic processing of endogenous Ala^1^-Ang II (Ang A) by ACE2 (62). Similar to Ang-(1–7), Ala^1^-Ang-(1–7) induced the relaxation of isolated aortic vessels and chronic infusion of the analog lowered blood pressure. Interestingly, the vascular effects of Ala^1^-Ang-(1–7) were not blocked by the typical receptor antagonist D-Ala7-Ang-(1–7) (A779) against the Mas receptor, but were attenuated by D-Pro^7^-Ang-(1–7) and the AT_2_ receptor antagonist PD123319. This study further showed that Ala^7^-Ang-(1–7) stimulated the Mas-related receptor (MrgD) and did not interact with the Mas receptor. Identification of Ala^1^-Ang-(1–7) in the human circulation and in an isolated heart perfusion system was achieved by a HPLC-Mass spectrometry approach. It is worth noting that the available direct RIA or ELISA assays will not distinguish between the Asp^1^- and Ala^1^- forms of Ang-(1–7) since both isoforms share the identical C-terminal sequence that is typically recognized by the immunoreactive antibodies. Thus, an initial separation step such as HPLC combined with conventional immunoreactive assays will be required to routinely detect and quantify the different forms of Ang-(1–7) in the circulation and tissues. The potential importance of these findings may reflect the greater diversity of the Ang-(1–7) axis regarding the identification of both a novel ligand and receptor that contributes to vascular tone. Moreover, that the AT_2_ antagonist PD12319 antagonized the actions of Ala^1^-Ang-(1–7) at the MrgD receptor may explain the apparent interaction of Ang-(1–7) with the AT_2_ receptor noted in several studies (63–65).

Although distinct from either Ang-(1–7) or its Ala analog, Jankowski et al. identified another endogenous ligand to the AT_7_/Mas receptor in human serum termed angioprotectin (66). This peptide resembles the octapeptide Ang II but has substitutions of Pro and Glu at the first two N-terminal residues to form Pro^1^-Glu^2^-Ang II. Despite the fact that the angioprotectin contains both the Tyr^4^ and Phe^8^ residues considered to be essential to the actions of Ang II, the peptide lacked any vasoconstrictor activity in isolated aortic rings. However, the peptide induced a dose-dependent vasorelaxation of isolated vessels that was absent in vessels from the Mas-knockout mice, as well as acutely reduced blood pressure in the SHR. Moreover, Pro^1^-Glu^2^-Ang II stimulated NO formation in Mas-transfected CHO cells but not in the control cells. Finally, the study presented evidence for local formation of Pro^1^-Glu^2^-Ang II from Ang II in human endothelial cells that was enhanced by addition of exogenous proline and glutamic acid suggesting a post-transcriptional modification of Ang II. It is not known to what degree Pro^1^-Glu^2^-Ang II is processed by ACE2 or other carboxypeptidases to the Ang-(1–7) analog and whether Pro^1^-Glu^2^-Ang-(1–7) is functionally active at the either the Mas or MrgD receptors. It is also unclear the extent conventional immunoreactive assays for Ang II will detect endogenous Pro^1^-Glu^2^-Ang II in plasma or tissues given their identical C-terminal sequence. The circulating levels of Pro^1^-Glu^2^-Ang II were 15% of Ang II in humans, but the Ang II analog increased fivefold in patients with end-stage renal disease that may perhaps reflect a compensatory response in pathological conditions (66).

### Ang-(1–7) metabolism

The endogenous levels of Ang-(1–7) are influenced by access to processing enzymes such as the carboxypeptidase ACE2 or the endopeptidases neprilysin, thimet oligopeptidase, and prolyl endopeptidase (oligopeptidase). The levels of Ang-(1–7) are also dependent on peptidases that metabolize the peptide. Similar to bradykinin and substance P, ACE plays a significant role in the hydrolysis of Ang-(1–7) to the pentapeptide Ang-(1–5) in the circulation and the proximal tubules of the kidney cortex (Figure 2) (22, 67). ACE inhibition increased the half-life of Ang-(1–7) sixfold in the circulation and is necessary to demonstrate the accumulation of Ang-(1–7) from both Ang I- and Ang II-dependent pathways in the renal proximal tubules (67, 68). Thus, the mechanism for the increased levels of Ang-(1–7) following ACE inhibitor treatment reflects both protection of the peptide from ACE hydrolysis to Ang-(1–5) and shunting of Ang I to Ang-(1–7) through endopeptidase pathways such as neprilysin or thimet oligopeptidase (23). There is relatively little information on other peptidases that participate in the metabolism of Ang-(1–7) other than ACE. We recently detected an endopeptidase activity in the cerebrospinal fluid (CSF) of sheep that metabolized Ang-(1–7) at the Tyr^4^-Ile^5^ bond to yield Ang-(1–4) and constituted the majority of Ang-(1–7) degrading activity in CSF (69, 70). Although the identity of the peptidase is currently unknown, the activity was insensitive to inhibitors against neprilysin, thimet oligopeptidase, or neurolysin (EC3.4.24.26) (70). The Ang-(1–7) peptidase activity was abolished by the mercury-compounds *p*-chloromercuribenzoate (PCMB) and aminophenyl-mercuriacetate (APMA), as well as the chelating agents *o*-phenanthroline and EDTA, but not the cysteine epoxide inhibitor E-64 suggesting a metallopeptidase-like activity in CSF (70). The regulation of the CSF peptidase is described in the proceeding section on fetal programing.

## Intracellular Ang-(1–7)-Mas Receptor System

The RAS was traditionally viewed as an endocrine system whereby circulating renin catalyzes an enzymatic cascade to form active peptide products; however, it is apparent that multiple tissues contain the necessary components for the local generation of angiotensin peptides (71, 72). These tissue systems may release the precursor angiotensinogen, the intermediate products Ang I and Ang-(1–12), or the active peptides Ang II and Ang-(1–7) to bind directly to cell surface receptors in an autocrine or paracrine manner. Robertson and Khairallah reported over 40 years ago the localization of Ang II binding sites on the chromatin fraction of vascular smooth muscle cells and cardiomyocytes suggesting an intracellular site of action for Ang II (73). Several laboratories subsequently identified Ang II receptors using classical receptor binding techniques on nuclei isolated from liver (74–76). Eggena and colleagues demonstrated that Ang II stimulated mRNA transcripts for angiotensinogen, renin, and PDGF from isolated liver nuclei suggesting that the nuclear binding sites were functional and capable of directly mediating gene expression (77, 78). Moreover, AT_1_ receptors were also evident on nuclei isolated from cortical and medullary areas of the rat kidney (79–81). Ang II-AT_1_-receptor stimulation on isolated renal nuclei increased mRNA expression of angiotensinogen, the sodium-hydrogen exchanger (NHE3) and the cytokine monocyte chemoattractant protein (MCP-1) (79). Ang II also elicited an immediate increase in calcium by isolated cortical nuclei or via microinjection of the peptide in intact epithelial cells (82). We find that Ang II directly stimulates reactive oxygen species (ROS) as demonstrated by the enhanced fluorescent signature of dichlorofluorescein (DCF); ROS formation was sensitive to the NAD(P)H oxidase inhibitor diphenyleneiodonium (DPI) and the AT_1_ antagonist losartan (83). Blockade of phosphoinositol 3-kinase (PI3K) and protein kinase C (PKC) abolished the Ang II-AT_1_-receptor-dependent stimulation of ROS in renal nuclei. In lieu of the nuclear localization of the NAD(P)H oxidase isoform NOX4, activation of AT_1_ receptors may acutely stimulate ROS by a PI3K-PKC pathway and subsequent phosphorylation of NOX4 (Figure 4) (83–87).

The studies demonstrating nuclear AT_1_ receptors within the kidney and other tissues clearly support an emerging view for the localization of various G-protein coupled receptors (GPRCs) to the nucleus (88–94). In regards to the Ang-(1–7)-Mas receptor system, O’Dowd and colleagues noted a canonical nuclear localization sequence on the Mas protein in their studies on AT_1_-receptor trafficking and localization in vascular smooth muscle cells (95). We undertook a series of studies to establish an intracellular role for Ang-(1–7) in the cortical tissue and proximal tubules isolated from the sheep kidney. Immunoblot analysis of nuclei isolated from sheep proximal tubules demonstrated a single immunoreactive band of 35 kDa utilizing an affinity-purified antibody against the human Mas protein (96). Receptor binding studies with the non-selective antagonist ^125^I-(Sarcosine^1^, Threonine^8^)-Ang II (Sarthran) revealed significant competition by the AT_7_/Mas receptor antagonist D-Ala^7^-Ang-(1–7) in nuclei isolated from the renal cortex. Functional assessment of the nuclear AT_7_ receptor was then assessed with the sensitive NO fluorophore DAF in the presence or absence of the NO synthase inhibitor L-NAME. Ang-(1–7) dose-dependently increased the fluorescent signature for NO which was abolished by prior treatment with L-NAME or the Ang-(1–7) antagonist, but not antagonists to the AT_1_ or AT_2_ receptors. Consistent with the stimulation of NO by Ang-(1–7), protein expression for endothelial nitric oxide synthase (eNOS) and soluble guanylate cyclase (sGC) was evident in the isolated nuclei of sheep proximal tubules (96). These data further support previous studies that localized eNOS and sGC to liver nuclei, as well as the stimulation of NO and cGMP by activation of the bradykinin B2 receptor (90, 97). The exact function of the Ang-(1–7) axis of the RAS within the nucleus is not known; however, we hypothesize this system may antagonize the intracellular actions of the Ang II-AT_1_-receptor pathway. To address this possibility, we assessed the influence of the selective ACE2 inhibitor MLN4760 and the Mas receptor antagonist on the activation of ROS by Ang II in renal cortical nuclei. The Ang II-AT_1_-receptor dependent increase in ROS was significantly augmented to a similar extent by treatment of nuclei with either the ACE2 inhibitor or the AT_7_ receptor antagonist (98). That both MLN4760 and D-Ala^7^-Ang-(1–7) increased the stimulation of ROS suggests that the conversion to Ang-(1–7) by ACE2 antagonizes the actions of the Ang II-AT_1_-receptor axis on the nucleus. It is possible that that simply blocking the degradation of Ang II with the ACE2 inhibitor may augment the actions of Ang II; however, the comparable effects of the AT_7_ receptor antagonist D-Ala^7^-Ang-(1–7) suggests a distinct role for Ang-(1–7). Since the Ang-(1–7) antagonist is a peptide and may potentially interact with ACE2, we further demonstrated that D-Ala^7^-Ang-(1–7) does not inhibit nuclear ACE2 activity as assessed by the HPLC-based conversion of Ang II to Ang-(1–7). Moreover, our studies suggest that the processing of Ang II to Ang-(1–7) by ACE2 on the nuclear membrane leads to the activation of signaling pathways distinct from that of Ang II (98). We do not know, however, whether the attenuation of ROS production by Ang-(1–7) involves the stimulation of NO or other signaling pathways. As previously discussed, Ang-(1–7) may attenuate the actions of Ang II and other growth hormones by the activation of intracellular phosphatases such as the dual specificity phosphatases MKP-1 and SHP-1 (50, 51). Several classes of phosphatases including MKP-1 traffic to the nucleus; however, it is unknown whether Ang-(1–7) can influence these enzymes to attenuate the actions of Ang II (99).

**Figure 3:**
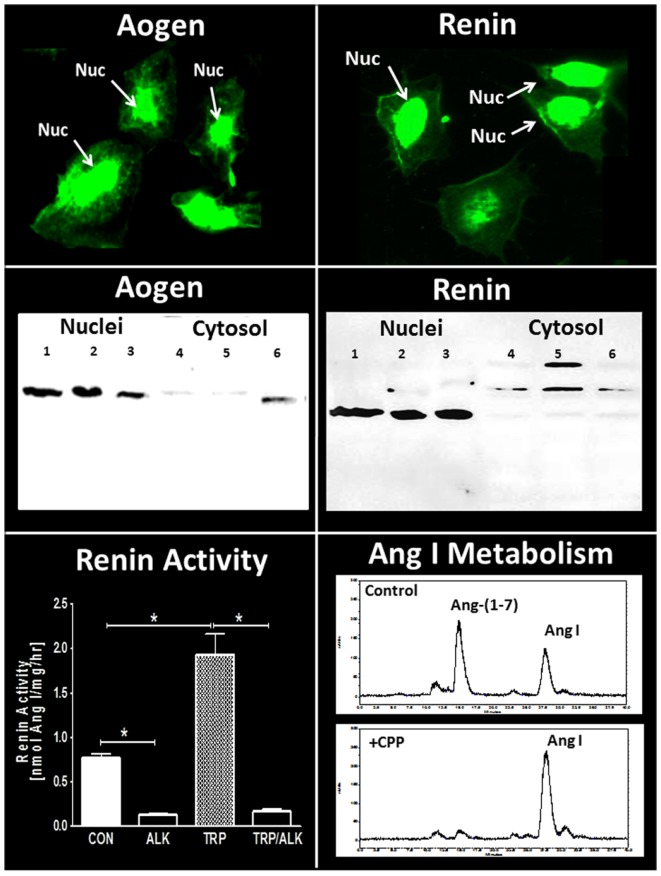
**Expression of intracellular components of the renin-angiotensin system in NRK-52E renal epithelial cells**. Immunofluorescent (IMF) staining and protein immunoblot for rat angiotensinogen (Aogen) and renin. Immunoblots of Aogen and renin in nuclei (lanes 1–3) and cytosol (lanes 4–6) were from three separate passages of NRK-52E cells. Major bands for Aogen and renin were identified at approximately 55 kDa. Renin activity (conversion of Aogen to Ang I) in isolated nuclear fractions was increased threefold following activation by trypsin (TRP) and was essentially abolished by the renin inhibitor aliskiren (ALK). Conversion of ^125^I-Ang I to ^125^I-Ang-(1–7) in the isolated nuclear fraction was predominantly blocked by the thimet oligopeptidase inhibitor CPP. Renin activity data are mean ± SEM; *n* = 4; *P < 0.05. Ang I metabolism representative of data from *n* = 4 separate cell passages. Adapted from Alzayadneh and Chappell (107).

Clearly, one issue regarding the intracellular RAS and other peptidergic systems is the localization of the components within the cell. The nucleus is composed of two distinct bilayers termed the outer (OMN) and inner (INM) nuclear membranes. Nuclear pore proteins traverse both membrane domains and facilitate transport between the cytosol and the nuclear matrix which contains the chromatin-DNA complex. Portions of the ONM are continuous with the endoplasmic reticulum (ER) such that perinuclear space is shared with the ER. The nuclear envelope comprising both OMN and INM invaginates into the nuclear matrix creating a nuclear reticulum that is key in the regulated release of nuclear Ca^2+^ (100–102). Although various studies have localized GPRCs primarily to the nuclear envelope and matrix, it is currently unclear how the peptide ligands target the nuclear GPRCs, as well as the precise coupling of the receptors to their signaling pathways within the nucleus. Moreover, elucidation of the pathways that deliver peptide ligands to their respective intracellular receptors, as well as the intracellular regulation under normal and pathological conditions has not been established. As to the intracellular expression of angiotensins in the kidney, there is evidence for expression and uptake of angiotensinogen, as well as the uptake of Ang II and Ang-(1–7) by protein transporters such as megalin (71, 103–106). In addition, AT_1_-receptor mediated internalization of Ang II may contribute to the intracellular content of the peptide (72, 89). In this regard, intracellular peptidases such as ACE2 may potentially process the internalized Ang II to Ang-(1–7) as alternative pathway to attenuate AT_1_-receptor activity and stimulate the cellular actions of Ang-(1–7). Utilizing the renal epithelial NRK-52E cell line, we find evidence for the nuclear localization of angiotensinogen (Figure 3, left panels) consistent with earlier findings by Sherrod and colleagues regarding nuclear angiotensinogen in brain astrocytes and isolated nuclei of sheep proximal tubules (96, 107, 108). Interestingly, a second antibody directed to the Ang I sequence of angiotensinogen failed to detect the protein in the nucleus of the NRK-52E cells suggesting that enzymatic processing of the precursor may occur in this compartment (107). In support of an intracellular processing pathway, renin expression was also evident in the nucleus of the NRK cells (Figure 3, right panels). Isolated nuclei exhibited both renin and prorenin activity (following activation by trypsin) that was sensitive to the specific renin inhibitor aliskiren (Figure 3, bottom left panel), as well as immunoreactive levels of Ang II and Ang-(1–7) (107). In addition, peptide metabolism studies in isolated nuclei revealed the direct conversion of Ang I to Ang-(1–7) that was essentially abolished by a selective inhibitor (CPP) of the metalloendopeptidase thimet oligopeptidase (Figure 3, bottom right panel). Others have reported the nuclear expression of thimet oligopeptidase in brain, as well as the identification of a nuclear localization sequence for the human peptidase (109, 110). The NRK-52E cells may constitute a relevant cell model to establish the pathways that contribute to the intracellular generation and actions of Ang II and Ang-(1–7) within renal epithelial cells. As an alternative concept to intracellular formation, Ibarra and colleagues presented evidence for another model of nuclear signaling whereby the plasma membrane invaginates to the perinuclear area that facilitates presentation of intracellular signals (IGF receptor coupled to IP_3_ formation) discretely to the nucleus in cardiomyocytes (111). The apparent advantages of this system may reflect a more selective activation of the signaling cascade and independence from the intracellular generation of the peptide ligands (112). The latter study adds another potential mechanism to the complex pathways of the intracellular receptor system for angiotensins and other peptides, as well as emphasize the need for additional studies to elucidate their organization and function.

In the endeavor to elucidate the intracellular pathways, the importance of robust biochemical and molecular techniques to characterize the RAS cannot be overly emphasized. Several reports have raised concerns regarding the specificity of commercial AT_1_ and AT_2_ antibodies widely utilized for western immunoblot and immunocytochemical distribution studies (113–115). Importantly, these studies find that receptor protein bands at the appropriate molecular weights were not abolished in AT_1_- or AT_2_-deleted cell and tissue samples. We have utilized antibodies to both AT_1_ and AT_2_ receptors to establish their molecular weight in the nuclear fraction as this pertains to the maturation or processing of the receptor protein. However, studies by our laboratory and others also incorporate peptide binding assays to quantitate receptor density and affinity, as well as various antagonists to identify the receptor subtype. The receptor binding assays also parallel the demonstration of functional signaling pathways (ROS, NO) on nuclei and the sensitivity to receptor antagonists. Reliance on the assessment of mRNA for the receptor may not equate to protein expression and certainly does not reveal the discrete intracellular distribution of the receptor. Antibodies to angiotensin receptors or other RAS components are useful and convenient tools to characterize this system; however, parallel approaches to establish the expression and regulation of the RAS particularly within the cell are clearly warranted.

## Fetal Programing

**Figure 4:**
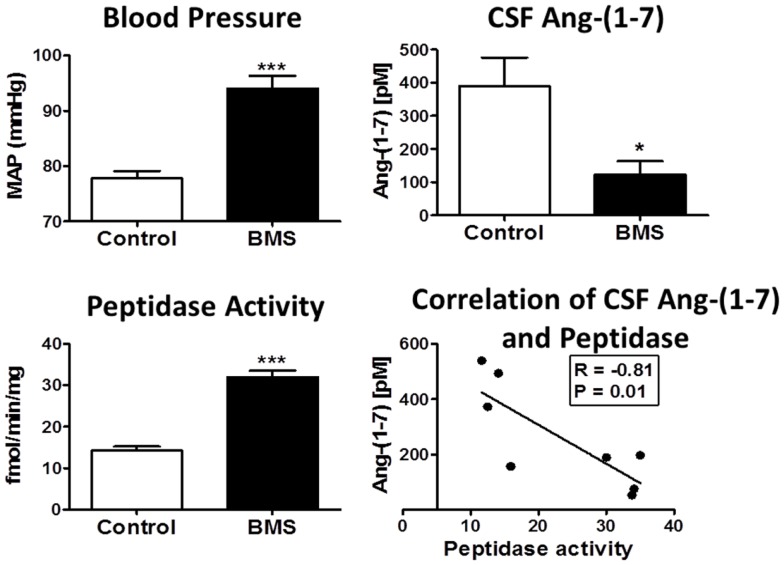
**Betamethasone-exposed (BMS) offspring exhibit higher mean arterial pressure (MAP) and CSF endopeptidase activity than non-exposed sheep**. Blood pressure (MAP) was higher in BMS animals at 6 months of age. CSF peptidase activity was twofold higher in BMS animals as compared to controls. CSF Ang-(1–7) peptide levels were lower in BMS animals. Ang-(1–7) peptide levels negatively correlate with peptidase activity in the CSF (*r* = −0.81, *P* = 0.01). Data are mean ± SEM; 4–5 per group; *P < 0.05 or ***P < 0.001 vs. controls. Adapted from Marshall et al. (70).

Increasing evidence for the influence of early prenatal events in the fetus to induce a greater susceptibility to cardiovascular and metabolic pathologies is evident in both experimental models and in humans (116–119). Although the precise nature of fetal programing events is not known, alterations in the biochemical components and functional aspects of the RAS may constitute an important underlying mechanism (69, 120–128). Our recent studies utilize a sheep model of fetal programing in which pregnant ewes are administrated the glucocorticoid betamethasone at day 80 of gestation. This regimen parallels the dose and time that pregnant women are typically treated with glucocorticoids to enhance pulmonary function and reduce mortality of the fetus delivered preterm. Fetal exposure to glucocorticoids in sheep results in a significant reduction in the nephron number within the kidney, an increase in mean blood pressure, attenuation of the baroreflex response (BRS) in the control of heart rate and increased indices of metabolic dysfunction in adult animals (129) (Figure 4). In regards to the function of the RAS following glucocorticoid exposure, acute treatment with the AT_1_-receptor antagonist candesartan normalized blood pressure in the exposed sheep and improved the impaired BRS, but had no overall effect on pressure in the control or unexposed adult sheep (129). In contrast, administration of D-Ala^7^-Ang-(1–7) increased blood pressure and attenuated BRS in the control but not the betamethasone-exposed (BMS) sheep suggesting that the loss of Ang-(1–7) tone may be an additional consequence of fetal programing events (126). The protein expression of the AT_7_/Mas receptor was significantly lower in the brain medulla in both 6-month- and 1.8-year-old BMS sheep as compared to age-matched control sheep; however, the AT_1_-receptor protein expression was unchanged (122). We also find reduced CSF levels of Ang-(1–7) in the exposed sheep, as well as higher activities of the Ang-(1–7) peptidase (Figure 4) (69, 70). Indeed, the CSF content of Ang-(1–7) inversely correlated to Ang-(1–7) peptidase activity in the control and BMS sheep (Figure 4, lower right panel). Thus, the reduced expression of the AT_7_/Mas receptor and increased metabolism of Ang-(1–7) in brain may contribute to the loss of Ang-(1–7) tone in BMS sheep, as well as the enhanced responsiveness of the Ang II-AT_1_-receptor pathway in glucocorticoid-dependent programing without significant changes in the AT_1_-receptor levels.

In regards to the renal RAS in fetal programing, Ang-(1–7)-dependent stimulation of sodium excretion was abrogated in the BMS sheep. Moreover, the anti-natriuretic response to Ang II was enhanced in the BMS sheep, as well as the reduction in renal plasma flow (130, 131). Consistent with the altered renal responses to Ang II and Ang-(1–7), expression of ACE2, the peptidase that contributes to the balance of Ang II to Ang-(1–7), was significantly reduced in the circulation, the proximal tubules and the urine of the BMS adult sheep (125). That both tubular and urinary forms of ACE2 were reduced in the BMS sheep suggests down regulation or reduced synthesis of the peptidase in the proximal tubules that may lead to the lower release or shedding of the enzyme from the apical membrane. Both ACE and neprilysin activities were readily detected in the proximal tubules and urine of adult sheep; however, their activities were not changed following betamethasone exposure. Moreover, circulating ACE activity increased while ACE2 activity decreased in the serum of BMS adult sheep (125). A kinetic analysis of ACE2 activity revealed a reduction in the maximal velocity (*V*_max_) of the enzyme rather than a change in substrate affinity (Km) suggesting reduced protein content in the circulation. These data further suggest that the soluble forms of the enzyme in serum exhibits similar kinetic characteristics as the native form, at least regarding the metabolism of Ang II to Ang-(1–7). The ratio of ACE to ACE2 also closely correlated with the mean blood pressure values in the control and BMS sheep (125).

In addition to the altered expression of ACE2, the balance of angiotensin receptor subtypes was changed as well (132). Moreover, the proportion of both AT_2_ and Mas receptor subtypes were lower in the renal cortex of the exposed group. However, the AT_1_ subtype was the predominant angiotensin receptor in the renal medulla and the receptor subtypes were unchanged between the control and exposed sheep. These data again emphasize the selective effects of fetal glucocorticoid exposure on expression of the RAS components within different tissue compartments. The intracellular studies on the Ang II and Ang-(1–7) signaling pathways further support the overall findings that the expression of angiotensin receptor subtypes were altered following glucocorticoid exposure. In isolated nuclei from the real cortex, the generation of NO by Ang-(1–7) was markedly reduced in the exposed sheep. In contrast, the Ang II-dependent stimulation of ROS via the AT_1_ receptor was augmented in renal nuclei from the BMS sheep. Furthermore, addition of the AT_2_ antagonist PD122319 exacerbated the production of ROS by Ang II, and this augmented response was particularly evident in the glucocorticoid-exposed group (132). It is not clear whether the AT_2_ receptor exhibits functional antagonism of the AT_1_ receptor to influence ROS formation or that the AT_2_ subtype sequesters Ang II from the AT_1_ receptor given the higher ratio of AT_2_ to AT_1_ sites in the sheep cortex.

Finally, fetal programing events may convey greater sensitivity to an additional stressor or insult, particularly as these animals age (119). Therefore, recent studies ascertained the renal responses to Ang-(1–7) in control and glucocorticoid-exposed adult sheep following removal of one kidney. In contrast to the intact, non-exposed sheep, Ang-(1–7) infusion reduced sodium excretion in uni-nephrectomized animals (133). The anti-natriuretic response to Ang-(1–7) was enhanced by the AT_7_ receptor antagonist D-Ala^7^-Ang-(1–7) and was subsequently blocked by the AT_1_ antagonist candesartan. Moreover, the exposed animals still exhibited an attenuated natriuretic response to the combination of Ang-(1–7) and candesartan in comparison to the unexposed control group. Similar hemodynamic responses were also observed for Ang-(1–7) in the uni-nephrectomized animals whereby the peptide alone reduced blood flow and the combination of Ang-(1–7)/candesartan increased flow; however, the overall vascular responses were similar between the control and exposed animals (133). Certainly, differences in species and sex, the primary or immortalized status of the cells, the dose of both peptide and antagonists, and the duration of treatment may influence the functional actions of Ang-(1–7) and the receptor(s) mediating these effects (134). In regards to the studies in the uni-nephrectomized sheep, the differential response in sodium handling to Ang-(1–7) likely reflects the compensatory mechanisms of the remaining kidney to accommodate the marked increase in cardiac output and fluid handling. The fascinating aspect of the renal studies is the plasticity in the Ang-(1–7) response that apparently encompasses an AT_1_ receptor interaction to reduce sodium excretion in the uni-nephrectomized animal, as well as the Mas receptor interaction to stimulate sodium excretion in the intact sheep. Moreover, the natriuretic response of Ang-(1–7) potentially mediated by the Mas receptor remains intact in the uni-nephrectomized sheep since it was unmasked by blockade with the AT_1_-receptor antagonist. However, the mechanism underlying the functional interaction of Ang-(1–7) with AT_1_ receptors in the single kidney is not known, as well as whether Ang-(1–7) stimulates signaling pathways identical to that of the Ang II-AT_1_-receptor axis to reduce sodium reabsorption and renal blood flow.

## Sex Differences in the Ang-(1–7)-Mas Receptor Axis

Both experimental and clinical evidence suggest an important influence of sex on the development of cardiovascular disease that may reflect the regulation of the RAS by gonadal hormones including testosterone and estrogen. Women are generally thought to be protected from cardiovascular pathologies up to the time of menopause suggesting a beneficial effect of estradiol; however, several large clinical trials utilizing estrogen or combined estrogen/progesterone replacement in older women with underlying cardiovascular disease revealed adverse effects of either treatment. Experimental studies have largely focused on the role of estrogen to influence the ACE-Ang II-AT_1_-receptor axis of the RAS and generally reveal an inhibitory effect on the expression of ACE and the AT_1_ receptor (135–138). Estrogen depletion by ovariectomy in young mRen2.Lewis rats markedly exacerbated the hypertension and essentially abolished sex differences in blood pressure between the male and female congenics (139). In this model, estradiol replacement or treatment with the AT_1_-receptor antagonist olmesartan normalized blood pressure suggesting the loss of estrogen may lead to the dysregulation of the RAS. Indeed, circulating levels of Ang II and ACE activity were higher in the estrogen-depleted mRen2.Lewis while plasma levels of Ang-(1–7) were reduced, and the overall ratio of Ang II to Ang-(1–7) increased (139, 140). Brosnihan and colleagues originally proposed that the protective effects of estrogen may, in part, reflect a shift in the balance between circulating Ang II and Ang-(1–7) that may arise from the inhibitory effects of the steroid on ACE to promote Ang-(1–7) expression via increased synthesis and/or reduced metabolism of the peptide (141).

**Figure 5:**
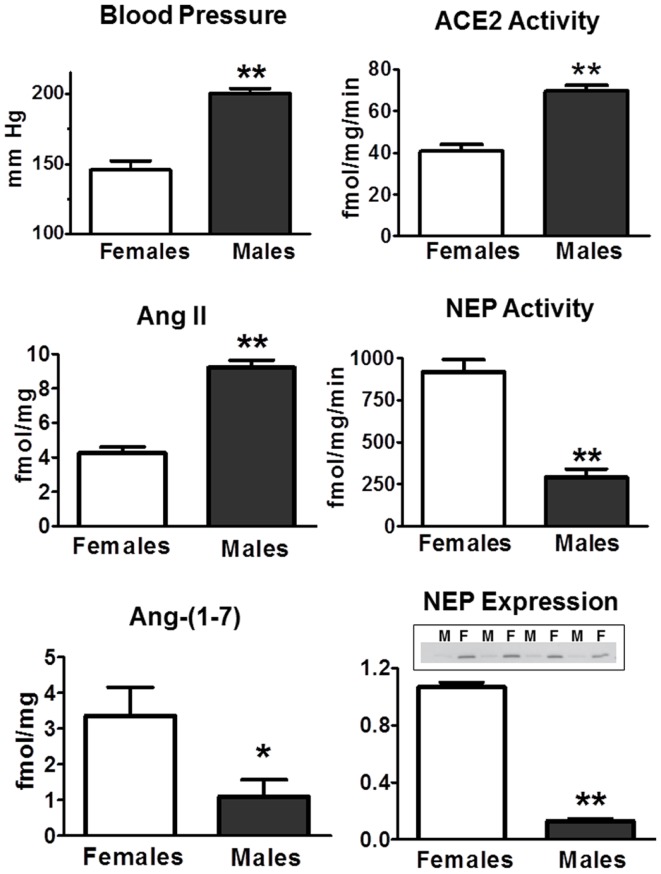
**Sex differences in systolic blood pressure and RAS components in the renal cortex of 15-week-old hemizygous mRen2.Lewis congenic rats**. Systolic blood pressure (mmHg) is higher in males. Intrarenal concentrations (femtomole peptide per milligram protein – fmol/mg) of Ang II are higher in males, but Ang-(1–7) content is lower. ACE2 activity (femtomole product per milligram protein per minute – fmol/mg/min) is higher in males, but neprilysin (NEP) activity is lower in males. NEP expression assessed by Western blot was lower in males (M) as compared to females (F). Data are mean ± SEM; *n* = 4–8 per group; *P < 0.05 or **P < 0.01. Adapted from Pendergrass et al. (81).

There are relatively few studies that have assessed tissue differences in Ang II and Ang-(1–7) in males and females. In the kidney of the mRen2.Lewis hypertensive rats, the tissue content of Ang II was twofold higher in the males (Figure 5). Conversely, renal levels of Ang-(1–7) were threefold lower in the males as compared to females. Interestingly, cortical ACE2 activity was 70% higher in the males perhaps suggesting a compensatory effect to buffer the higher Ang II content and blood pressure evident in the male mRen2.Lewis (Figure 5). In contrast, cortical neprilysin activity and protein expression were significantly higher in the female congenics as compared to males. The higher content of the endopeptidase may contribute to the differential expression of angiotensins in the female kidney to favor the enhanced conversion of Ang I to Ang-(1–7), as well as the metabolism of Ang II to Ang-(1–4). Cardiac ACE2 activity was also significantly higher in the male congenics; however, tissue levels of Ang II and Ang-(1–7) were not different between males and females. The renal content of Ang-(1–7) was also higher in female SHRs as compared to the males; however, tissue levels of Ang II were not different (142). Although this study did not determine peptidase expression in the SHR kidney as a potential mechanism for the higher Ang-(1–7) content, tissue levels of Ang-(1–7) were higher in females following Ang II treatment perhaps suggesting greater processing of Ang II to Ang-(1–7). Sandberg and colleagues assessed sex differences in ACE2 expression in the mouse kidney using the “four core” approach to distinguish the effect of sex chromosomes and ovarian steroids (143). Consistent with the results in the mRen2.Lewis rats, renal ACE2 activity and expression were higher in the male mice (143). Ovariectomy increased ACE2 expression in the female kidney, and estradiol replacement reduced the peptidase in both males and females; however, there was no influence of gonadal steroids on either cardiac or pulmonary ACE2 (143). These data suggest that at least under non-pathological conditions, estrogen exhibits an inhibitory influence on kidney ACE2 and raises the issue of whether this response contributes to the deleterious effects of estrogen replacement in older women. Cassis and colleagues reported the sex-dependent expression of circulating Ang-(1–7) in mice fed a high fat (HF) diet (144). Males exhibited a marked decline in plasma levels of Ang-(1–7) that was associated with higher circulating Ang II; however, plasma levels of Ang-(1–7) increased in the HF-fed females. Interestingly, ovariectomy of the HF-fed female mice reduced circulating Ang-(1–7) and ACE2 activity in adipose tissue but did not influence renal ACE2. The fall in circulating Ang-(1–7) was associated with a marked increase in nocturnal blood pressure. Moreover, administration of D-Ala^7^-Ang-(1–7) increased blood pressure in female mice maintained on the HF diet suggesting that the ACE2-Ang-(1–7)-Mas axis may buffer obesity-induced hypertension to a greater extent in females.

In regards to the Ang-(1–7) receptor, expression of the Mas receptor was increased in females but not in males following the infusion of Ang II which may explain the attenuated blood pressure to Ang II in the females (142). Pretreatment with the AT_7_ receptor antagonist D-Ala^7^-Ang-(1–7) enhanced the blood pressure response to Ang II in the female SHR. Similar findings were recently reported in the aldosterone salt-sensitive model where the mRNA levels for both ACE2 and the Mas receptor increased 1.5- and 5-fold, respectively, in the lamina terminalis (LT) of female rats (145). Chronic ICV treatment with the D-Ala^7^-Ang-(1–7) antagonist markedly augmented the blood pressure response in intact females treated with aldosterone and sodium chloride. Moreover, ovariectomy exacerbated the blood pressure response to aldosterone/salt; however, the mRNA expression of ACE2 or the Mas receptor in the LT area was not changed suggesting an inability to upregulate the central Ang-(1–7) axis contributes to the increase in blood pressure in this model (145). Denton and colleagues reported that D-Ala^7^-Ang-(1–7) alone decreased renal blood flow in female but not male normotensive Wistar rats, although the antagonist did not influence the renal response to acute Ang II infusion in either sex (64). In a separate study, the mRNA levels of the Mas protein were markedly higher in the kidney of adult female rats as compared to the males (146). Interestingly, Mas expression tended to decline in the male kidney while the mRNA levels of the receptor increased in females over the postnatal period (1–110 days). Our studies in the sheep model of fetal programing also provide evidence for sex differences in the responsiveness to Ang-(1–7). Although the exposure to betamethasone results in a similar increase in blood pressure and reduction in nephron number in the male and female sheep, the renal response to Ang-(1–7) or the antagonist differs with sex. An acute infusion of Ang-(1–7) results in a robust natriuretic response in control females but not the adult males (130). Moreover, betamethasone exposure was associated with reduced natriuresis in the males with or without Ang-(1–7) treatment, but significantly blunted the natriuretic actions of Ang-(1–7) in females. At this time, the mechanism underlying the sex-dependent effects of Ang-(1–7) on sodium excretion in control and betamethasone-exposed sheep are not known, but we speculate that it may involve the altered expression or signaling of the Mas receptor on the tubular elements of the kidney.

## Summary

The current review has examined several aspects from the recent literature on the non-classical or alternative ACE2-Ang-(1–7)-Mas receptor axis of the RAS. The mounting biochemical and functional evidence clearly supports the tenet that this pathway may antagonize the ACE-Ang II-AT_1_-receptor arm of the RAS either directly through metabolism of Ang II to Ang-(1–7) by ACE2 or via distinct pathways that limit the activation of Ang II-AT_1_-receptor signaling. Indeed, the demonstration of an intracellular ACE2-Ang-(1–7)-Mas axis that attenuates the Ang II-dependent stimulation of ROS on renal nuclei is in keeping with the concept of a balanced RAS even within the cell and emphasizes the importance of targeting the intracellular system as a therapeutic approach to enhance the functional ratio of Ang-(1–7) to Ang II. The evidence that an altered Ang-(1–7) system within the brain and the kidney following antenatal glucocorticoid exposure implicates an interaction between the Ang II and Ang-(1–7) pathways that contribute or promote the cardiovascular dysfunction associated with fetal programing events. Finally, sex differences apparent in blood pressure regulation and cardiovascular pathologies may reflect alterations in the ACE2-Ang-(1–7)-Mas receptor axis of the RAS in addition to those effects typically associated with the ACE-Ang II-AT_1_-receptor pathway.

## Conflict of Interest Statement

The authors declare that the research was conducted in the absence of any commercial or financial relationships that could be construed as a potential conflict of interest.
